# Can mouse kidney transplant models inform mechanisms of injury and acceptance in clinical kidney transplantation?

**DOI:** 10.1016/j.ajt.2025.04.001

**Published:** 2025-04-09

**Authors:** Zheng Jenny Zhang, Federica Casiraghi, Griffith Boord Perkins, William M. Baldwin, Robert L. Fairchild

**Affiliations:** 1 Comprehensive Transplant Center, Northwestern University Feinberg School of Medicine, Chicago, Illinois, USA; 2 Department of Surgery, Northwestern University Feinberg School of Medicine, Chicago, Illinois, USA; 3 Istituto di Ricerche Farmacologiche Mario Negri Istituto di Ricovero e Cura a Carattere Scientifico (IRCCS), Bergamo, Italy; 4 Adelaide Medical School, The University of Adelaide, Adelaide, South Australia, Australia; 5 Department of Inflammation and Immunity, Lerner Research Institute, Cleveland, Ohio, USA; 6 Cleveland Clinic Lerner College of Medicine, Cleveland, Ohio, USA

**Keywords:** kidney transplantation, mouse models, surgical approaches, rejection, tolerance

## Abstract

Despite standard-of-care immunosuppression, acute rejection remains commonly observed in kidney transplants and leads to chronic graft injury and failure in many transplanted patients. Mechanisms underlying acute and chronic kidney graft injury are incompletely understood, undermining the development and implementation of therapeutic strategies to improve outcomes. This compels the use of preclinical kidney transplant models to identify components and mechanisms mediating acute and chronic graft injury. Mouse models have been instrumental in establishing basic principles of alloimmune responses and the rejection of heart allografts. There is increasing use of mouse models to extend these studies to kidney transplantation, but the relevance of the findings to clinical kidney transplants remains under scrutiny. Here, we discuss the strengths and weaknesses of mouse models of kidney allograft responses and injury. Although obvious weaknesses arise when considering the relevance to clinical kidney transplants, there are new models that recapitulate many features of kidney graft injury in the clinical scenario and have much to contribute to understanding innate and donor alloantigen-specific mechanisms underlying kidney allograft injury. As in most preclinical studies, the pertinent use of kidney allogeneic transplants in mice comes down to the judicious choice of test questions and the choice of appropriate donors and recipients for the chosen model.

## 1. Introduction

Kidney transplantation is currently the optimal cure for endstage kidney disease and the highest volume transplant performed clinically. Allogeneic kidney transplants may be subject to several different causes of innate and adaptive immune injury, including ischemia-reperfusion injury, T cell–mediated rejection, and antibody-mediated rejection (ABMR), which remain important risk factors for poor graft outcomes. Mechanisms underlying these responses remain far from being understood and compel the use of preclinical models to delineate their initiation and progression in promoting kidney allograft injury and failure. Mouse models have been key tools in identifying the components and interactions that lead to T cell and antibody responses to pathogens, cancer, and autoantigens, as well as the roles of innate immunity in mediating tissue inflammation and initiating antigen-specific responses. Mouse transplant models have elucidated the fundamentals of allogenicity and roles of T cells and antibodies in allograft rejection.^1–3^

Skin and heterotopic cardiac transplantation have emerged as preferred models to study alloimmune responses and graft rejection; however, the growing appreciation for physiological relevance has highlighted the benefits of kidney transplant models. The ability to evaluate experimental interventions based on kidney allograft function and survival ensures that findings from these models are definitive and pertinent, with analogous readouts of graft function (eg, serum creatinine, urea, proteinuria, and/or glomerular filtration rate) directly applicable to clinical studies.

## 2. Kidney transplant surgery in mice

Mouse kidney transplantation can be life-sustaining or nonlife-sustaining.^3–5^ To generate a life-sustaining model, both recipient’s native kidneys are removed and the donor’s kidney is connected to the urinary tract. The life-supporting model can be performed using either a 1-stage technique in which bilateral nephrectomy of the native kidneys is performed at the time of transplant or a 2-stage procedure in which 1 recipient native kidney is left intact at transplant and on day 4 to 7 posttransplant is removed or rendered nonfunctional by imposing an acute ureter obstruction. The technical variances associated with these 2 techniques can significantly influence allograft functional recovery and the kinetics of host alloimmune responses to the allograft.

The decision on which approach to use depends on both the specific research purpose and the microsurgeon’s experience and preference. The standard 1-stage procedure, though technically demanding, renders the clinically relevant situation where the recipient is entirely dependent on the transplanted kidney function. With a skilled microsurgeon, the 1-stage procedure can be performed with a 90% success rate. The 2-stage procedure allows sufficient time for the kidney graft to recover from ischemia-reperfusion injury and provides an opportunity to directly observe or biopsy the graft during the second surgery. However, exposing animals to 2 major surgeries and additional anesthesia can increase the risk of complications from the surgical trauma and inflammation. Moreover, the presence of a normal native kidney may alter graft perfusion and tissue regeneration, influencing the host donor-reactive response, depending on when the delayed nephrectomy is performed.^3^ Studies in rats have demonstrated that the temporary retention of a fully functioning native kidney increases macrophage infiltrates and shortens the survival of the allograft.^6,7^ Nonlife-sustaining models in which the contralateral native kidney is left in situ for the duration of the experiment have been utilized to great effect for studies focusing on allograft histopathology. To this end, 1 study of complete MHC-mismatched CBA allografts transplanted to C57BL/6 recipients did not find obvious differences in rejection histopathology at day 7 with and without an in situ native kidney despite a 2- to 3-fold increase in serum creatinine and a 5- to 6-fold increase in serum urea in the latter.^8^

As the field looks to improve the relevance and translatability of findings from mice, the life-sustaining model has become the accepted standard for murine kidney transplantation. The dependency on graft viability may also improve the physiological relevance of the kidney transplant model as the recipient experiences the effect of kidney failure. C57BL/6 recipients with a long-term surviving BALB/c donor kidney show a progressive rise in serum creatinine reaching 3- to 4-fold higher levels than isograft controls at day 100.^9^

Two methods are currently used for the restoration of urinary tract continuity: anastomosis between the donor ureter-bladder patch to the recipient bladder^5^ or direct insertion of the donor ureter into the recipient bladder.^10^ The technique for ureter-bladder patch to bladder anastomosis remains the preferred method for many microsurgeons. Although ureter to bladder insertion may be a quicker, easier procedure to perform, it is associated with an increased risk of urinary complications, including urine leakage.

Consistency in the age and gender of donors and recipients as well as surgical techniques are keys to achieving high reproducibility of any model. For technical purposes linked to size, 10- to 14-week-old mice, weighing 25 to 30 grams, are preferred as both kidney graft donors and recipients. When using the delayed nephrectomy approach it is important to be consistent with the time/date of the second surgery.

## 3. How kidney allograft rejection is defined in mouse models

Rejection is primarily defined by evaluating histological lesions on tissue sections stained with hematoxylin and eosin and periodic acid-Schiff, similar to clinical practice. Acute rejection is graded based on the prevalence and severity of interstitial lymphocyte infiltration, tubulitis, glomerulitis, and arteritis—key morphological signs of acute rejection in clinical kidney transplantation. Each lesion is scored from grade 0 (no changes) to 3 (>75%) according to the percentage of parenchymal involvement, quantified in distinct, nonoverlapping fields of the renal cortex and medulla. These grades can be adapted to the Banff classification system as follows: 0 = normal, 1 = borderline change, 2 = IA, 3 = IB, 4 = IIA, 5 = IIB, 6 = III.^5^ Interstitial mononuclear cell infiltration is typically the first lesion observed, peaking at 7 to 10 days posttransplant. These infiltrates either stabilize or regress by day 21 posttransplant without immunosuppression in many strain combinations as discussed below.^11^ During the first posttransplant week, T cell–associated tubulitis, arteritis, and glomerulitis are minimal but progressively develop, becoming severe by day 21 resembling acute rejection in human kidney allografts. These observations and recent single-cell transcriptomic analyses in mouse kidney transplant models provide critical mechanistic insights into the roles of T cells and macrophages in driving acute rejection of kidney allografts.^12^

Kidney allografts surviving initial acute cellular rejection develop histopathological lesions resembling clinical chronic rejection abnormalities over time with increased glomerular mesangial and endocapillary cell hyperplasia, capillary loop thickening, and severe interstitial fibrosis—hallmarks of chronic rejection in humans.^13^ Severe interstitial fibrosis and tubular atrophy have also been observed long-term using congenic murine strains with a single class II mismatch, H-2^bm12^ kidney into C57BL/6 recipients.^14^ About 40% to 90% BALB/c kidneys transplanted to C57BL/6 recipients survive long-term (>100 days) in the absence of immunosuppression, with the surviving grafts declining functionally and developing severe glomerulosclerosis, interstitial fibrosis and tubular atrophy with time, similar to the progressive fibrosis observed in clinical kidney transplants in the absence of clinical rejection.^13,15,16^

The progressive development of acute and chronic kidney allograft injury can be followed by measuring serum creatinine or urea nitrogen levels in blood or N-GAL in the urine. A novel approach that is easily performed and reproducibly measures transdermal glomerular filtration rate in mice has also been recently reported.^17^ Mice living in speciic pathogen free cage conditions and without impetus to actively move can survive with a minimal number of nephrons making serum creatinine measurements a poor assessment of kidney transplant injury in mouse models. Multiparametric functional MRI avoids the need for euthanizing animals for histological analysis. MRI parameters (acquired using a 7-Tesla small animal MRI scanner) were compared with kidney histology in mice that received allogeneic or syngeneic kidney grafts at 3 weeks and 6 weeks posttransplant.^18,19^ A stronger impairment of renal perfusion, higher T2 and T1 relaxation times, and lower apparent diffusion coefficient were observed in allogeneic vs syngeneic grafts. These imaging parameters correlated with cellular infiltration (F4/80^+^ and CD4^+^ T cells) and the degree of interstitial fibrosis assessed by fibronectin staining. Renal T1 mapping and diffusion-weighted MRI are currently under investigation as promising noninvasive markers of fibrosis and graft function in humans.^20^ Another noninvasive monitoring technique uses micro-CT imaging, which allows high-resolution, real-time imaging of kidney graft location and function in live mice.^21^

## 4. Different alloimmune responses and kidney allograft rejection elicited in donor-recipient strain combinations

Immunosuppression noncompliance leads to acute T cell–mediated rejection in clinical transplantation. In contrast, mouse kidney transplants show a remarkable propensity for long-term immunosuppression-free survival and can be used to model a range of rejection phenotypes. The pattern of kidney allograft rejection and recipient survival in mouse models varies depending on the donor-recipient strain combinations.^3,4,22–24^ BALB/c and C57BL/6 strains are commonly used due to the availability of transgenic and knockout models on these backgrounds. C57BL/6 allografts transplanted to BALB/c recipients result in the majority (>70%) failing to survive beyond days 14 to 30 due to mixed acute cellular and vascular rejection, with less than 30% of the recipients surviving beyond day 100.^25^

Because long-lived allografts in these models experience early T cell–mediated inflammation, they are well-suited for studies of cell populations infiltrating and persisting in the allograft. Use of a transgenic semiallogeneic (BALB/c × B6)F1 → C57BL/6 kidney model demonstrated that recipient dendritic cells within the graft maintain the direct alloimmune response and that infiltrating T cells persist in the allograft as bona fide resident memory cells and drive chronic rejection.^26,27^ For a similar effect, C57BL/6 recipients of fully allogeneic BALB/c kidney allografts treated daily with 1 mg/kg intraperitoneal tacrolimus implicated graft-infiltrating macrophage differentiation into myofibroblasts as a key contributor to chronic interstitial fibrosis.^28^ Murine models have also proven useful for the study of kidney-trophic viruses in transplantation^29–33^ and studies of drugs and biomaterials that target specific attributes of the kidney.^34,35^

## 5. Development of donor-specific antibodies in mouse kidney transplant models

A major barrier in clinical transplantation is preformed anti-HLA antibodies in patients sensitized through prior blood transfusion, transplant, or pregnancy. This can be modeled in mice by presensitization with a skin graft; eg, presensitization of C57BL/6 recipient mice with a skin graft from DBA/2 mice results in a shift from spontaneous tolerance to failure of DBA/2 kidney allografts within 10 days of transplant.^36^ These mice exhibit high titers of circulating anti-MHC antibodies and histological lesions indicative of mixed cellular and antibody-mediated rejection. The observed ABMR fulfills all clinical criteria of humoral rejection as follows: (1) clinical evidence of graft dysfunction, (2) histological evidence of tissue injury, (3) complement activation split product deposition in peritubular capillaries, and (4) the presence of donor-specific antibodies (DSA). Mixed cellular and antibody-mediated rejection has also been observed after skin graft presensitization in other strain combinations.^37,38^ Passive transfer of DSA to immunodeficient kidney allograft recipients recapitulates aspects of acute ABMR, including deposition of C4d on glomerular and peritubular capillaries that is accompanied by endothelial cell swelling, capillary dilation, platelet activation, and margination of monocytes.^39^

Despite being a major cause of graft loss in the clinic, spontaneous formation of de novo DSA is not a major feature of most mouse kidney transplant models. To investigate the donor-reactive T cell compartment in de novo DSA responses, alloreactive memory CD4^+^ T cells were isolated from donor A/J or BALB/c skin-sensitized C57BL/6 mice for adoptive transfer into naïve C57BL/6 mice and upon kidney transplantation, these mice, which lacked preformed antibodies, displayed accelerated rejection characterized by high serum DSA titers, diffuse C4d deposition in peritubular capillaries and glomeruli, and histological evidence of vascular changes associated with antibody-mediated injury (eg, peritubular capillary dilation, endothelial cell swelling, and margination of monocytes), without significant T cell infiltration.^40^ In unmanipulated mouse models, antibody responses result in various degrees of C4d deposition and evidence of endothelial cell injury, which can be augmented by prolonged cold ischemia of the donor kidney before transplantation.^41^

Transplantation of complete MHC-mismatched kidneys to B6.CCR5^–/–^ recipients results in acute rejection of all allografts within 18 to 28 days.^42^ CCR5 deficiency is associated with dysregulated CD4^+^ T cell development skewed to Th2 cells. CCR5-deficient mice receiving kidney allografts develop high titers of DSA inducing histopathological changes identical to clinical acute ABMR, including margination of neutrophils and macrophages in tubular capillaries, double contouring of glomerular capillaries, and diffuse C3d deposition. This rejection is accompanied by the appearance of serum markers of kidney graft injury, deposition of C3d and C4d in the graft vasculature, and expression of transcripts diagnostic of clinical kidney graft ABMR that include transcripts associated with natural killer (NK) cell activation.^43,44^ This model has revealed the role of interdependent graft-infiltrating NK cell and myeloid cell activation that is initiated following DSA binding to the graft endothelium.^44,45^ Moreover, absent NK cell activation abrogates acute ABMR despite the high serum DSA titers and leads to prolonged graft survival with the development of chronic ABMR that includes interstitial fibrosis, glomerulopathy, and arteriopathy observed in clinical kidney transplants.^43–45^

## 6. The kidney as a factor in tolerance and rejection

Several of the strain combinations that result in the long-term survival of kidney allografts generate acute rejection of cardiac and skin allografts. The distinct biological and physiological features of kidneys may account, at least in part, for the differential survival of kidney allografts observed in the different donor→recipient combinations.^22,46–49^ Kidneys of C57BL/6 mice display more severe histological and functional impairment than kidneys of C3H or BALB/c mice, regardless of recipient strains or alloreactivities, suggesting that donor kidney-intrinsic factors contribute to the severity of graft injury. Damage to a portion of kidney graft tissue may not significantly impact the function of other nephrons and life may be sustained by partial kidney function, at least temporarily.

Beyond delayed graft failure, certain strain combinations result in bona fide operational tolerance that can extend to donor-matched skin or cardiac transplants and be adoptively transferred with T cells from the tolerant recipient to naïve mice just before transplantation with a kidney allograft,^50,51^ including DBA/2→C57BL/6,^23,52^ A/J→C57BL/6,^36^ and C57BL/6→B10.BR.^49^ These models have identified periarterial aggregates of FOXP3^+^ regulatory T cells (Tregs) infiltrating accepted allografts and revealed Treg function, including TGFb expression and development of indoleamine 2,3-dioxygenase–producing dendritic cells within the graft.^23,47,53–55^ Treg depletion dissolves these aggregates and abrogates tolerance.^54^ Notably, adoptive transfer of the graft-infiltrating Tregs transferred donor alloantigen-specific tolerance to naïve mice.^53^ The potential translational relevance of kidney-derived erythropoietin in the development of these infiltrating Tregs in mice was demonstrated in a prospectively controlled clinical study, which showed expansion of Tregs with erythropoietin use in patients with chronic kidney disease.^56^

These regulatory infiltrates are now being reexamined as Treg-rich organized lymphoid structures or TOLS.^57^ In mice, TOLS are observed in accepted kidney allografts where they are associated with de novo lymphatic formation, and their composition and function have been the subject of recent single-cell transcriptomic studies.^12,58^ Although the role of TOLS in clinical transplants is yet to be elucidated, lymphatic vessels within human allograft biopsies were associated with better 1-year allograft survival, suggesting the translational relevance of mouse tolerance mechanisms.^59^ Forced lymphangiogenesis in kidney allografts from a doxycycline-inducible VEGF-C over-expressing donor better recapitulated the lymphatic vessels observed in human allograft TOLS, and delayed graft rejection 2.5-fold in an aggressive rejection model, C57BL/6/129S1 → BALB/c.^60^ Conversely, proinflammatory TOLS present in the donor kidney were sufficient to cause rejection of haplotype-mismatched F1 allografts in BALB/c recipients that lacked secondary lymphoid organs.^61^ Other potential underlying factors impacting kidney allograft responses include the size or mass of the organ to be transplanted, but these remain to be more thoroughly investigated.^62^

## 7. Inducing tolerance

In mice, tolerance is defined as indefinite graft survival and the acceptance of a secondary graft from the donor but not from a third-party source. As discussed above, tolerance to a kidney allograft can develop spontaneously in mice, with Tregs playing a dominant role in preventing the pathogenic donor-reactive T cell response. In murine kidney transplantation, tolerance can be achieved through the establishment of mixed chimerism, costimulatory blockade (eg, CTLA4-Ig and anti-CD154 mAb), or Treg induction/infusion.^63^ These approaches leverage the same natural mechanisms that maintain tolerance to self, including clonal deletion of autoreactive T cells in the thymus or peripheral mechanisms inducing deletion, anergy, or active suppression by regulatory T cells. The transfer of donor hematopoietic stem cells to the recipient to induce mixed chimerism (ie, the coexistence of donor and recipient hematopoietic cells), enables donor antigen-presenting cells to populate the recipient thymus and induces clonal deletion of developing donor-reactive T cells.^64^ However, this model requires a conditioning regimen to both reset the host immune system and allow space for donor hematopoietic stem cell engraftment, similar to the mixed chimerism achieved in clinical kidney transplant recipients.

To demonstrate that tolerance has been achieved, the tolerant recipient should accept a secondary graft from the same donor (eg, a skin graft) while rejecting a graft from a third-party source, proving the donor-specificity feature of tolerance.^65^ If donor-specific Tregs are formed, lymphocytes should transfer the donor-specific tolerance to naïve recipients—a phenomenon known as infectious tolerance.^66^

## 8. Limitations and improving the model

Although kidney grafts in mice offer a model of a life supporting transplant, there are several differences with clinical transplants. Some of these are inherent to biological divergences in the 2 species and others relate to technical disparities in the procedures and require critical consideration when interpreting the clinical implications of experimental data.

The basic protocol used in mice of removing a kidney from a healthy donor and immediately transplanting it to a healthy recipient most closely simulates a preemptive transplant from a living donor to a recipient who has not yet required dialysis. This elemental protocol excludes the physiological stresses endured by deceased donors, preservation injury to the organ during transportation, and the detriments of dialysis to the recipient awaiting a transplant. Pratschke et al^67^ mimicked the deleterious effects of brain death including “cytokine storm” by inflating catheters that were inserted into the brains of rats. Expression of TNF-a and IL-1b transcripts increased while the donors were maintained on mechanical ventilation, and kidney allografts from brain-dead donors underwent accelerated rejection with increased neutrophil and macrophage infiltrates. Similar methods have since been applied to mice.^68^ Graft preservation injury has been investigated by subjecting the kidney to various lengths of cold ischemic storage before transplantation or delaying the removal of vascular clamps after completing the vascular anastomoses to the recipient. The effects of imposing 4 hours of cold preservation in the absence of alloimmune responses have been tested in recipients of isografts and found to be dependent on genetic differences in innate signaling pathways.^69^ In the setting of allografts, 6 hours of cold ischemic storage has increased anti-MHC class II, but not anti-class I MHC, antibodies.^70^

Another technical difference that deserves investigation is the location of the transplant. In humans, kidneys are transplanted heterotopically to the lower abdomen with a short ureteral connection to the bladder. In smaller animals, kidneys are transplanted to the upper abdomen where the aorta and vena cava have a larger caliber. This location requires a longer segment of the ureter to reach the bladder and inflammatory processes related to rejection in allografts, not in isografts, result in dysfunction of the ureter and consequent hydronephrosis that compounds chronic rejection of the kidney. A similar complication of ureteral rejection has been reported for porcine kidneys xenografted to cynomolgus monkeys.^71^

Various recipient comorbidities have received limited attention. Obesity, a frequent variable before and after transplantation in humans, is an intriguing area awaiting exploration in mice because of studies demonstrating metabolic effects on both innate and adaptive immunity as well as direct effects on the kidney.^17,72^ The effects of aging on the immune system and the inflammatory content of the transplanted kidney have been investigated in some mouse models.^62^ The aging process is multifactorial and single-cell techniques have uncovered similarities and distinctions between humans and mice of age-related changes in innate and adaptive immune cells.^73^

Among the many immunological differences between mice and humans, several are directly relevant to kidney transplants.^74^ These include differences in the expression of MHC class II molecules on renal vascular endothelium. HLA-DR and DQ are expressed constitutively at various levels on human glomerular and peritubular capillary endothelial cells.^75^ In contrast, MHC class II antigens are not expressed on kidney endothelial cells in healthy mice but can be rapidly induced by proinflammatory cytokines such as IFN-g.^70,76^

With the emphasis on studying ABMR, it is important to be aware that several commonly used mouse strains have complete or partial complement deficiencies.^77^ These strains should be avoided unless questions regarding specific complement components are being tested.

Approaches to better mimic recipient pretransplant disease and posttransplant comorbidities need more stringent development in the mouse models discussed above. Differences between mice and clinical transplant patients with respect to kidney graft recipient immune allo-recognition and function and to graft immune and physiological features need further genetically manipulated tools and thoughtful experimental design in mouse kidney transplant models to increase understanding and resolution of the many challenges confronted in clinical kidney transplantation.

## 9. In summary

Kidney transplantation mouse models have emerged as useful tools to investigate mechanisms underlying acute and chronic injury and the development of donor tolerance. The ability to follow the function and survival of kidney allografts allows serial monitoring of the injury, and the associated immune response, and affords the opportunity to identify graft and recipient biomarkers associated with the injury. There are several models where these aspects of the response mirror those observed in clinical kidney transplants. Moreover, the development of chronic injury is an important clinical development, and models of this pathology in kidney transplants in mice should expose new insights into mechanisms leading to the development of chronic injury. Further development of these chronic kidney allograft models should also lead to the identification of targets to inhibit and possibly reverse this insidious disease which is a major limitation in the long-term survival of kidney grafts and patients. Although the long-term acceptance of kidney allografts without immunosuppression in many donor to recipient mouse strain combinations does not reflect what is observed in the clinic, careful choice of the model can provide insights that have proven relevant to humans.

## Abbreviations:

ABMR: antibody-mediated rejection
DSA: donor-specific antibodies
NK: natural killer
Treg: regulatory T cells
TOLS: Treg enriched organized lymphoid structures
